# Ensemble learning techniques reveals multidimensional EEG feature alterations in pediatric schizophrenia

**DOI:** 10.3389/fnhum.2025.1530291

**Published:** 2025-08-07

**Authors:** Ying Mao, Fang Wang, Shan Wang, Zhaowei Wang, Gang Li, Xuchen Qi, Yu Sun

**Affiliations:** ^1^Department of Special Examination, Shaoxing People’s Hospital, Shaoxing, China; ^2^School of Medicine, Shaoxing University, Shaoxing, China; ^3^Department of Neurology, Shaoxing People’s Hospital, Shaoxing, China; ^4^College of Mathematic Medicine, Zhejiang Normal University, Jinhua, China; ^5^Department of Neurosurgery, Sir Run Run Shaw Hospital, Zhejiang University School of Medicine, Hangzhou, China; ^6^Department of Rehabilitation, Sir Run Run Shaw Hospital, Zhejiang University School of Medicine, Hangzhou, China; ^7^Key Laboratory for Biomedical Engineering of Ministry of Education of China, Zhejiang University, Hangzhou, China

**Keywords:** pediatric schizophrenia, electroencephalogram, ensemble learning, feature selection, brain function

## Abstract

Schizophrenia (SCZ) is a severe mental disorder that impairs brain function and daily life, while its early and objective diagnosis remains a major clinical challenge due to the reliance on subjective assessments. This study aims to develop a machine learning-based framework for the auxiliary diagnosis of SCZ using multi-dimensional electroencephalogram (EEG) features and to investigate the underlying neural alterations. Resting-state EEG data were obtained from 45 male patients with pediatric SCZ and 39 age-and gender-matched healthy controls. Three types of EEG features (relative power (RP), fuzzy entropy (FuzEn), and functional connectivity (FC)) were extracted under various time window lengths and fed into four ensemble learning models. A data-driven feature selection approach (Recursive Feature Elimination) was applied to identify the most informative features, resulting in 212 most discriminative features (48 RP, 40 FuzEn, and 124 FC) out of the initial 760. Leveraging the selected features, the Categorical Boosting model achieved the highest classification accuracy of 99.60% at the 4-s window. Further analysis of the discriminative features revealed that the altered EEG characteristics were mainly in the alpha, beta, and gamma bands. Particularly, altered FCs exhibited a fronto-increase-parieto-decrease pattern mainly in the right hemisphere along with spectral-dependent RP alterations and a universally reduced FuzEn in the pediatric SCZ group. In summary, this study not only showcases the potential of advanced ensemble learning algorithms in precisely identifying pediatric SCZ, but also provides new insights into the altered brain functions in pediatric SCZ patients, which may benefit the future development of automatic diagnosis systems.

## 1 Introduction

Schizophrenia (SCZ) is a severe mental disorder characterized by symptoms such as cognitive impairments, persistent hallucinations and delusions, which significantly affect patients’ daily functioning and quality of life (Hirano and Uhlhaas, 2021). According to the latest research estimates in 2024, SCZ affects approximately 0.32% of the global population (Oprea et al., 2024). There are approximately 24 million SCZ patients worldwide, making it one of the top 25 leading causes of disability (Rahul et al., 2024). SCZ typically onsets in late adolescence or early adulthood and can have a lasting impact throughout the patient’s life (Buckley and Miller, 2015). Currently, antipsychotic medications are the primary treatment for SCZ, with about 70% of patients experiencing symptom improvement through appropriate treatment. However, the long-term outcomes of the disease vary widely among individuals: about 25% of patients achieve good recovery, 50% have moderate disability, and 25% experience significant persistent symptoms throughout their lives (Ranjan et al., 2024). The mortality rate among SCZ patients is two to three times higher than that of the general population, and their average lifespan is ten to twenty years shorter than that of healthy individuals (Laursen, 2011). SCZ not only causes profound suffering to patients but also imposes a heavy burden on families and society. Therefore, early diagnosis and deeper understanding of the neural mechanisms of SCZ, especially for pediatric SCZ, are crucial for improving treatment outcomes, reducing the disease burden, and enhancing patients’ quality of life (Wang et al., 2024; Li et al., 2024).

EEG, as a non-invasive technique for monitoring neural electrical activity, has the advantages of low cost, high temporal resolution, portability, flexible experimental design, and strong real-time feedback ability (Pan et al., 2024), and has extensive application value in neuroscience and brain function research (Pan et al., 2025; Pei et al., 2025). SCZ, as a complex and highly heterogeneous mental disorder, EEG shows unique advantages in revealing its underlying neural mechanisms. Numerous studies have demonstrated significant EEG abnormalities in patients with SCZ (Perrottelli et al., 2021; Perrottelli et al., 2022; Grohn and Eriksson, 2022; Hamilton and Northoff, 2021). For instance, aberrations in EEG power have been associated with specific cognitive impairments, such as deficits in verbal learning and memory function in SCZ patients (Koshiyama et al., 2021; Tanaka-Koshiyama et al., 2020). The elevated or reduced power anomalies exhibited by these patients significantly correlate with cognitive dysfunction, reflecting disturbances and imbalances in brain activity (Hamilton and Northoff, 2021; Iglesias-Tejedor et al., 2022). Moreover, the irregularity and complexity of EEG signals as assessed by entropy exhibited abnormal patterns in EEG signals of SCZ patients both at rest and during cognitive tasks (Goshvarpour and Goshvarpour, 2022; Molina et al., 2020). These entropy anomalies not only indicate disrupted neural network synchronization within the brain but also directly relate to patients’ negative symptoms and impairments in verbal memory ability, indicating that entropy disturbances might be a core factor in cognitive decline and could potentially serve as a biomarker for assessing disease progression and treatment efficacy (Molina et al., 2020). Recent findings have revealed that SCZ is associated with functional dysconnectivity between disparate brain regions (Fornito et al., 2012). Studies have found significantly reduced connectivity between key functional areas such as the prefrontal cortex and temporal region, and this diminished connectivity may be closely linked to the manifestation of symptoms, including cognitive impairments (Naim-Feil et al., 2018). Additionally, EEG-based dynamic studies of brain networks have revealed altered dynamic patterns of brain network connectivity in SCZ patients during cognitive tasks, characterized by increased global efficiency, decreased clustering coefficients, and changes in connection strength within specific brain regions. These changes are particularly evident within specific time windows following cognitive stimulation, further reflecting dynamic imbalances in brain function (Sun et al., 2019; Yan T. et al., 2023). In sum, multi-dimensional EEG measures have emerged as potent tools for unveiling the underlying mechanisms of SCZ and provide profound insights into the pathophysiological basis of SCZ.

In recent years, machine learning techniques have played an increasingly pivotal role in the diagnosis of SCZ, particularly in the analysis of resting-state EEG signals, where they have demonstrated remarkable advantages (Perellón-Alfonso et al., 2023; Yan W. et al., 2023; Lin et al., 2023). Numerous studies has concentrated on extracting multi-dimensional features from EEG to precisely identify patterns of brain electrical activity associated with SCZ. These studies can be broadly categorized into two main directions. First, a significant body of research has focused on applying advanced machine learning algorithms, such as adaptive neuro-fuzzy inference systems and 3D convolutional neural networks, achieving high classification accuracies for SCZ at 99.92% (Najafzadeh et al., 2021) and 97.74% (Shen et al., 2023), respectively. These investigations not only validate the efficacy of machine learning in the interpretation of complex EEG signals but also lay a solid foundation for its application in SCZ diagnosis. Second, other studies’ efforts are directed towards extracting EEG features and integrating them with traditional machine learning or deep learning models to further enhance diagnostic accuracy and provide interpretable findings. For instance, a study based on brain functional connectivity analysis, which fused different connectivity measures combining Partial Directed Coherence and *PLI* features, attained an accuracy of 95.16% (Zhao et al., 2021). Another study combined three effective connectivity measures (partial directed coherence, direct directed transfer function, and transfer entropy) with convolutional neural networks and transfer learning, elevating the diagnostic accuracy to 96.67% (Bagherzadeh and Shalbaf, 2024). These achievements not only uncover specific alterations in brain functional connectivity and other features in SCZ patients but also provide new insights into optimizing machine learning for EEG feature extraction and disease diagnosis.

While EEG has shown great promise in SCZ research, most existing studies rely on single-dimensional features or focus solely on adult populations, limiting their ability to reveal comprehensive neural patterns. To address these gaps, this study proposed a systematic analytical framework to dissect the brain functional mechanisms in pediatric SCZ patients. The primary contributions of this paper are summarized as follows:

1. *Integration of multi-dimensional EEG features:* Univariate power spectrum, fuzzy entropy, and multivariate functional connectivity were extracted to capture spectral, nonlinear, and network-level characteristics of brain activity.
2. *Machine learning based feature selection and classification:* Ensemble learning algorithms were employed to identify the most informative feature subset, enabling accurate differentiation between pediatric SCZ patients and healthy controls.
3. *Revealing abnormal brain mechanisms:* Group-level analyses were conducted to uncover specific alterations in power, entropy, and functional connection, providing insights into the electrophysiological dysfunctions associated with pediatric SCZ.

## 2 Materials and methods

### 2.1 Participants

The data used in this study were publicly available from the Mental Health Research Center (MHRC), Russian Academy of Medical Sciences, including 45 boys diagnosed with schizophrenic disorders (infant SCZ, schizotypal and schizoaffective disorders corresponding to F20, F21 and F25 according to the ICD-10) and 39 age-matched healthy participants. The age of patients ranged from 10 years and 8 months to 14 years, while the healthy participants ranged from 11 years to 13 years and 9 months. The mean age of both groups is 12 years and 3 months. The diagnoses of the patients were performed and confirmed by specialists of the MHRC. None of the patients were undergoing chemotherapy during the examination period at the MHRC. Further details pertaining to the clinical characteristics of patients could be found in reference (Borisov et al., 2005). The current study with the objective of data analysis, was approved by the Institutional Review Board of the Shaoxing People’s Hospital.

### 2.2 EEG data recording and preprocessing

EEG data were recorded from 16 channels while the participants were in an awake and relaxed state with their eyes closed. The electrode positions (i.e., F3, F4, F7, F8, C3, C4, Cz, T3, T4, T5, T6, P3, P4, Pz, O1, and O2) were placed in accordance with the 10–20 international standard system. The reference electrode was the left and right mastoid and the sampling frequency was set as 128 Hz. A previously validated standard EEG preprocessing pipeline was adopted for raw EEG signals (Dimitrakopoulos et al., 2018), the specific preprocessing procedure included the following steps: (1) A bandpass filter was applied to filter the data to the 0.5–45 Hz range, in order to remove low-frequency drifts and high-frequency EMG interference; (2) The EEG signals were re-referenced using an average reference across all electrodes; (3) Fast ICA was used to extract independent components, and artifacts were identified with the help of manual inspection and the ICLabel tool, in order to remove non-neural artifacts such as eye movements, blinks, and muscle activity; (4) The same filter was used to further divide the signals into five standard frequency bands: Delta (0.5–4 Hz), Theta (4–8 Hz), Alpha (8–13 Hz), Beta (13–30 Hz), and Gamma (30–45 Hz); (5) The preprocessed and band-divided signals were segmented into six time windows of different durations, providing a foundation for the extraction of multidimensional EEG features across multiple temporal scales. All preprocessing steps were conducted using customized codes in MATLAB 2021b (The MathWorks, Inc., U. S.) and the EEGLAB toolbox (Delorme and Makeig, 2004).

### 2.3 Feature extraction

After obtaining the artifact-free preprocessed EEG data, three widely used features that cover linear/nonlinear univariate and multivariate domains were adopted in this work for feature extraction, including relative power spectrum (RP), fuzzy entropy (FuzEn), and phase lag index (PLI). The extracted features were subsequently used as inputs for the following machine learning models.

*Relative Power Spectrum* (*RP*): For a given EEG signal *x*(*t*) (*t* = 1, 2, 3, …, *N*; *N* is the time point of *x*(*t*)), its spectrum *x*(*f*) can be estimated using Fast Fourier Transform. The power spectrum *P_x_*(*f*) is then obtained via $P_{x}(f)=\frac{1}{N}|X(f)|2$ . Then, *RP* of EEG band can be estimated by Equation 1:


(1) $$\mathrm{RP}(h)=\frac{\int_{f_{l}}^{f_{h}}P_{x}(f)\mathrm{df}}{\int_{f_{n}}^{f_{m}}P_{x}(f)\mathrm{df}}\times \frac{f_{m}-f_{n}}{f_{h}-f_{l}}$$

where *f_h_* and *f_l_* are the upper and lower limits of different rhythms, and *f_m_* and *f_n_* are the frequency bounds of the EEG signal. The *RP* was estimated within each frequency band for each channel, resulting 16 × 5 *RP* features.

*Fuzzy Entropy* (*FuzEn*): For a given EEG signal *x*(*t*), it can be reconstructed into a set of *m*-dimensional vectors $X_{t}^{m}=[x(t),x(t+1),\cdots ,x(t+m-1)]-{\bar{x}}_{0}(t)$ , (*t* = 1, 2, …, *N*-*m* + 1), where *m* is the embedding dimension, ${\bar{x}}_{0}(t)$ is the mean value, and *N* is the length of the given signal *x*(*t*). Then the distance $d_{\mathrm{ij}}^{m}$ between two vectors $X_{i}^{m}\;$ and $X_{j}^{m}\;$ can be calculated as Equation 2:


(2) $$d_{\mathrm{ij}}^{m}=\max_{k\in (1,m)}\{|(u(i+k)-u_{0}(i))-(u(j+k)-u_{0}(j))|\},(i\neq j)$$

Then the *O_m_*(*r*) could be estimated by Equation 3:


(3) $$\begin{array}{c} O^{m}(r)=\frac{1}{N-m}\sum_{i=1}^{N-m}(\frac{1}{N-m-1}\sum_{j=1,j\neq i}^{N-m}\exp(-\ln(2)\cdot {\left(\frac{d_{\mathrm{ij}}^{m}}{r}\right)}^{2})) \end{array}$$

The *FuzEn* of the given signal *x*(*i*) could be obtained by Equation 4 (Al-sharhan et al., 2001):


(4) $$\begin{array}{c} \text{FuzEn}(m,r,N)=\ln O^{m}(r)-\ln O^{m+1}(r) \end{array}$$

In the current work, the embedding dimension *m* is set to 2, and *r* is determined by *k* × *δ*. Here, *k* is a constant value set to 0.2 (typically ranging between 0.10 and 0.25), and *δ* is the standard deviation of the EEG signal *x*(*i*). Within each frequency band, the *FuzEn* was estimated for each channel, leading to 16 × 5 features.

*Phase Lag Index* (*PLI*): *PLI* was adopted to estimate the functional connectivity for its superiority in minimizing the influence well-known volume conduction and common sources (Stam et al., 2007). For a given pair of two EEG signals *x_k_*(*t*) and *x_l_*(*t*), the instantaneous phase is calculated using the Hilbert transform as Equation 5:


(5) $$\begin{array}{c} \begin{array}{c} z_{k}(t)=x_{k}(t)+i{\tilde{x}}_{k}(t)=Z_{k}(t)e^{j\phi_{k}(t)}\\ z_{l}(t)=x_{l}(t)+i{\tilde{x}}_{l}(t)=Z_{l}(t)e^{j\phi_{l}(t)} \end{array}, \end{array}$$

where *Z_k_* and *Z_l_* are instantaneous amplitude, *ϕ_k_*(*t*) and *ϕ_l_*(*t*) are the instantaneous phases at *t* moment, ${\tilde{x}}_{k}(t)$ is the Hilbert transform of each time series. Then the *PLI* between these two signals could be defined as Equation 6:


(6) $$\begin{array}{c} \mathrm{PLI}(k,l)=|\frac{1}{N}\sum_{t=1}^{N}\mathrm{sign}(\phi_{k}(t)-\phi_{l}(t))| \end{array}$$

where ∣·∣ means the absolute value, and *sign* stands for the signum function as Equation 7:


(7) $$\begin{array}{c} \mathrm{sign}(x)=\{\begin{array}{c} 1\;\text{if}\;x>0\\ 0\;\text{if}\;x=0\\ -1\;\text{if}\;x<0 \end{array}\;\phi_{k}(t)-\phi_{l}(t)\in [0,2\pi ] \end{array}$$

The range of *PLI* values is from 0 to 1. A large *PLI* value indicates a strong degree of phase synchronization between the pair of EEG signals, e.g., *PLI* = 0 indicates no coupling while *PLI* = 1 means two signals are in complete phase synchronization. After the functional connections of all pairs of channels were estimated, a 16 × 16 *PLI* matrix was obtained for each frequency band. Given that *PLI*(*k*, *l*) = *PLI*(*l*, *k*), a total number of (16 × 15 / 2) × 5 *PLI* features were obtained.

### 2.4 Ensemble learning models

Once we obtained the EEG features, four widely-used ensemble learning models that were popular in classification studies of EEG signals were adopted here to assess the performance of pediatric SCZ identification, including Random Forest (RF), eXtreme Gradient Boosting (XGBoost), Categorical Boosting (CatBoost), and Light Gradient Boosting Machine (LightGBM).

RF is an ensemble learning method that enhances prediction accuracy and stability by combining multiple decision trees. Each tree is trained on a random subset of the data and selects features randomly at each split, which helps reduce the risk of overfitting. Ultimately, RF derives the final prediction by aggregating the predictions of all trees. It is widely used in data science due to its simple implementation, rapid training, and robustness to outliers and noise.

XGBoost is a powerful gradient boosting algorithm, highly regarded for its exceptional predictive performance. It constructs a series of weak learners (typically decision trees) to progressively reduce prediction errors and improve model accuracy. XGBoost incorporates regularization techniques to control model complexity and overfitting, while employing efficient column sampling and parallel processing strategies that significantly enhance training speed and predictive performance. Additionally, it can handle missing values and offers a variety of flexible parameter tuning options, making it excellent in practical applications.

CatBoost is an efficient ensemble learning framework particularly suitable for handling complex categorical feature data. It employs symmetric decision trees and ordered boosting techniques to reduce overfitting and enhance the model’s generalization ability. CatBoost automatically processes categorical features and missing data, simplifying the data preprocessing pipeline, and accelerates model convergence through adaptive learning rate adjustments, thereby demonstrating outstanding performance in various application scenarios.

LightGBM is a fast and efficient gradient boosting framework designed for large-scale datasets. It adopts a histogram-based learning approach and incorporates a series of optimization strategies, such as Gradient-based One-Side Sampling (GOSS) and parallel processing of leaf splitting, to improve training speed and prediction accuracy. LightGBM excels in handling high-dimensional features and big data, making it widely applicable across various domains.

Taking the 4-s time window as an example, the dataset contained a total of 1,260 samples, including 585 HC samples and 675 SCZ patient samples. A 10-fold cross-validation approach was employed as the model basis, where the dataset was randomly divided into ten subsets, with nine as the training set and the remaining one as the test set. As such, by iterating this procedure 10 times, we could obtain the average classification performance metrics, including accuracy, precision, Recall, and F1 score.

### 2.5 Feature selection

Recursive feature elimination (RFE) is a model-based feature selection algorithm designed to identify the most significant features for model prediction performance from a feature set (Yan and Zhang, 2015). It operates through an iterative process to progressively evaluate and eliminate features that contribute less to model performance, thereby achieving feature selection and model optimization. Initially, a baseline model is trained using all features, and importance scores are calculated for each feature. These importance scores are typically derived from model coefficients or the extent to which features influence prediction outcomes. Subsequently, features are ranked based on their importance, and those with the least contribution to the model are gradually eliminated. After each feature elimination, RFE retain the model and recalculates the importance scores for the remaining features. This process continues until a predetermined number of features remain or until there is no significant improvement in model performance. This stepwise elimination strategy effectively identifies the most predictive features while reducing the interference of redundant features, thereby enhancing model accuracy and stability. By systematically selecting features, RFE helps us to identify the most discriminative features. This approach not only improves the computational efficiency but also mitigates the risk of overfitting, thereby improving the model’s generalization ability. Heuristically, RFE provides a pure data-driven approach for quantifying the contribution of each feature to the final prediction outcomes and reveals the role of each feature within the overall model through feature importance ranking, thereby facilitating the interpretation of the etiology of SCZ.

## 3 Results

### 3.1 Classification performance

**Figure 1:**
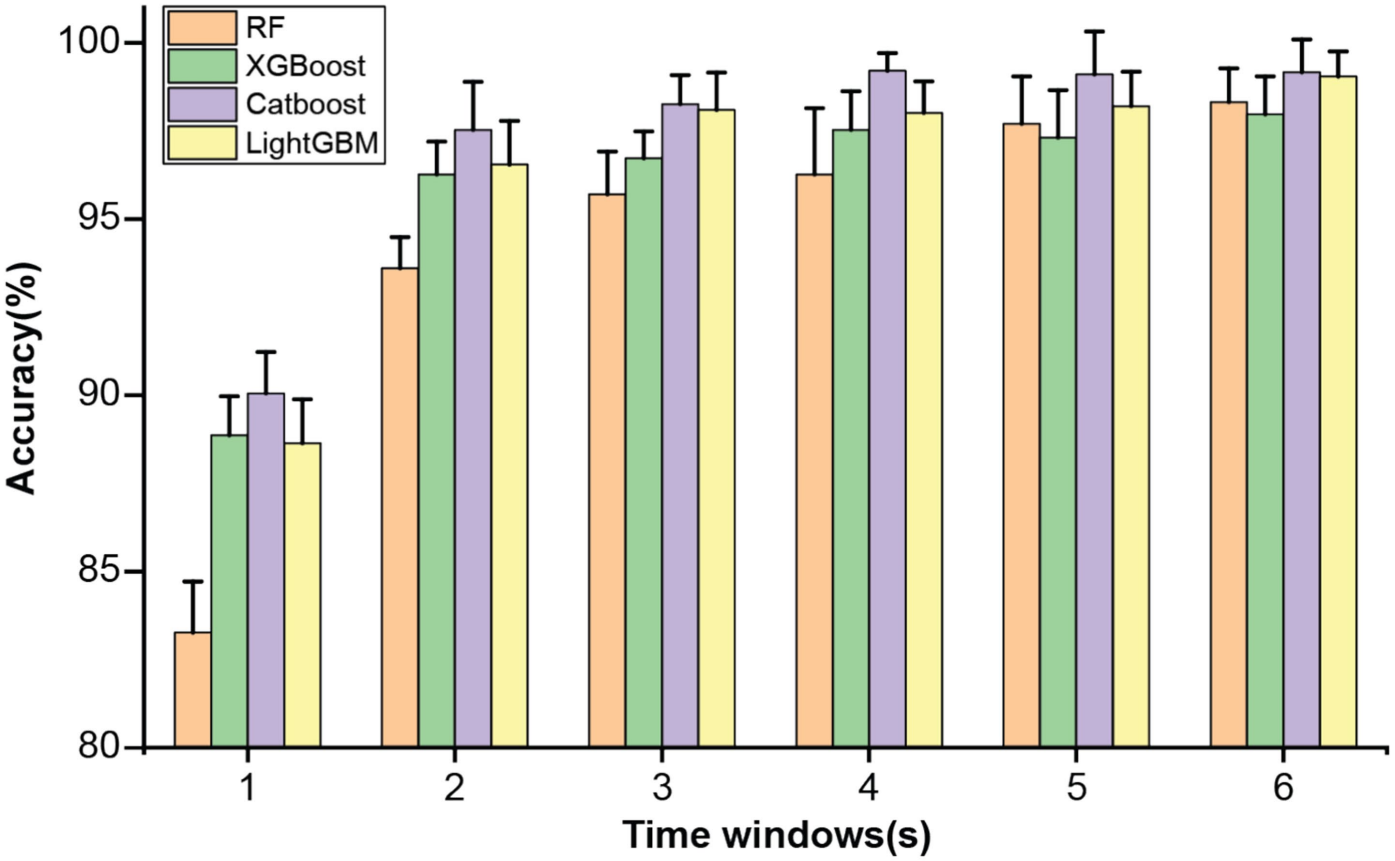
Classification accuracy of four ensemble learning models under different time windows.

The abovementioned multidimensional EEG features were estimated using a time window approach. To evaluate the influence of the window length on the classification performance, the proposed analysis framework was applied using six different window lengths: 1, 2, 3, 4, 5, and 6 s. Specifically, multidimensional EEG features were estimated within each non-overlapping window and set as input for the ensemble learning models. Figure 1 illustrates the classification performance of four ensemble learning models under six window lengths. As shown in Figure 1, longer window lengths led to improved estimation of EEG features, which in turn enhanced classification performance. All models achieved satisfactory results when the window length exceeded 2 s, while further increases in window duration produced only marginal improvements. The CatBoost classifier demonstrated the best classification performance with a 4 s time window (Table 1). Therefore, the window length of 4-s was used for the following analyses.

**Table 1:** Model performance results of the four ensemble learning models.

Models	Accuracy	Precision	Recall	F1 score
RF	96.27 ± 1.88	98.73 ± 1.43	93.15 ± 3.98	95.81 ± 2.27
XGBoost	97.54 ± 1.09	97.26 ± 2.32	97.43 ± 1.66	97.32 ± 1.26
CatBoost	**99.21 ± 0.50**	**99.30 ± 0.86**	**98.98 ± 0.83**	**99.14 ± 0.54**
LightGBM	98.02 ± 0.89	98.50 ± 1.11	97.22 ± 2.26	97.83 ± 1.01

Values are mean ± standard deviation. Bold indicates best performance.

### 3.2 Feature selection

**Figure 2:**
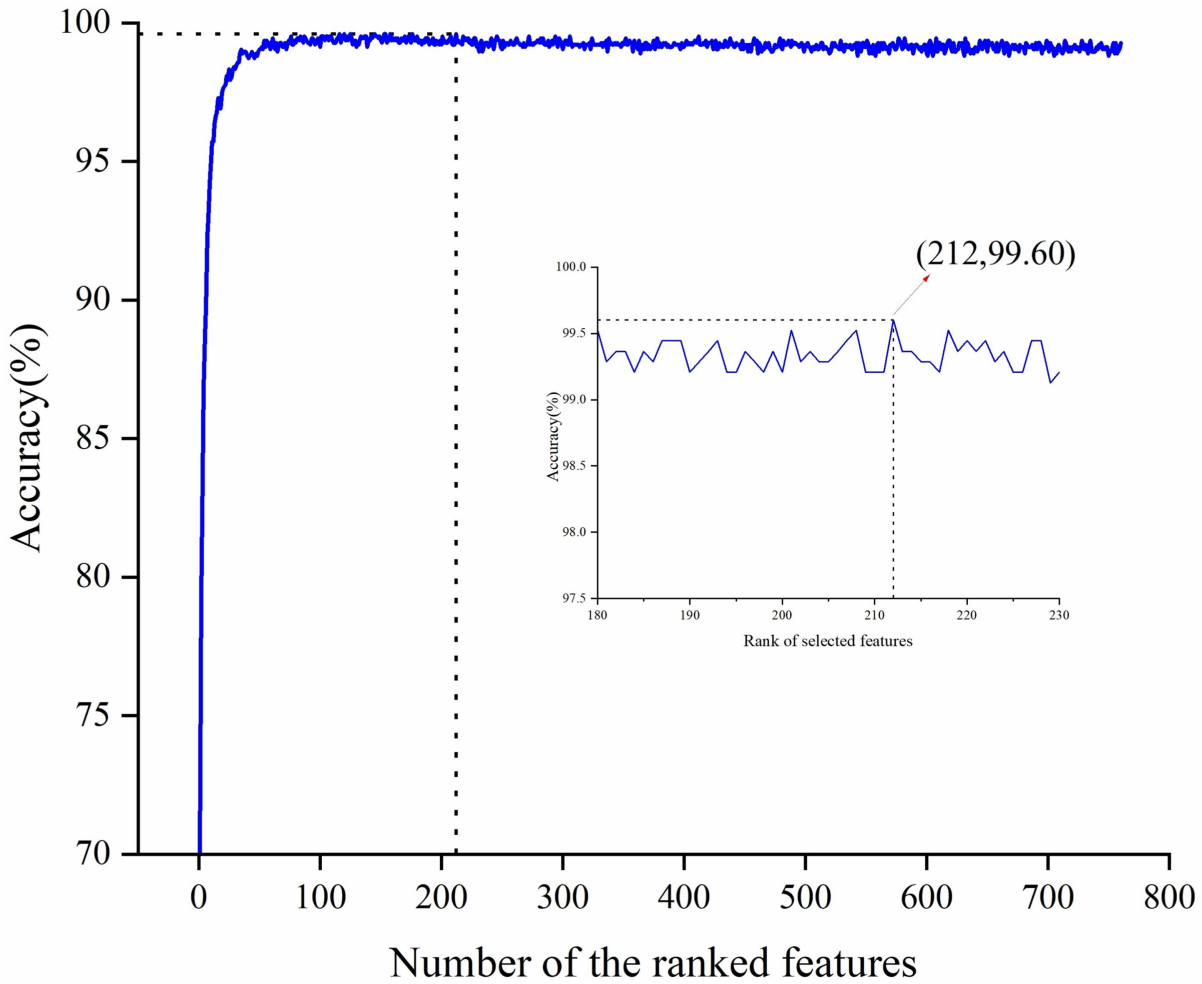
Classification accuracy during the RFE-based feature selection process.

Given that the full feature set comprises 760 features, potentially leading to feature redundancy, this study conducted feature selection based on the entire feature set. Considering the superior performance of CatBoost, it was chosen as the base model for RFE. By applying the RFE algorithm to the CatBoost model, the contribution scores were obtained for all features and then sorted in ascending order, thereby determining the feature importance ranking. Subsequently, the least contributive features were iteratively removed based on their importance and the CatBoost model was retrained by adding one feature per cycle until all features were traversed. The accuracy peaked at 99.60% when the number of input features for the model was 212. The variation of its accuracy rate with the number of features is shown in Figure 2. Therefore, the top 212 features with the highest contribution scores were selected as the optimal feature subset. We then further interrogated the frequency distribution of the obtained optimal feature subset and found a predominance toward high frequency bands (Delta /Theta /Alpha /Beta/Gamma = 12/31/54 /64/51) (Table 2).

**Table 2:** Feature distribution of the optimal feature subset.

Feature type	Delta	Theta	Alpha	Beta	Gamma	Total
RP	6	5	14	11	12	48
FuzEn	0	6	10	10	14	40
PLI	6	20	30	43	25	124
Total	12	31	54	64	51	212

### 3.3 Spatio-spectral distribution of the discriminative features

**Figure 3:**
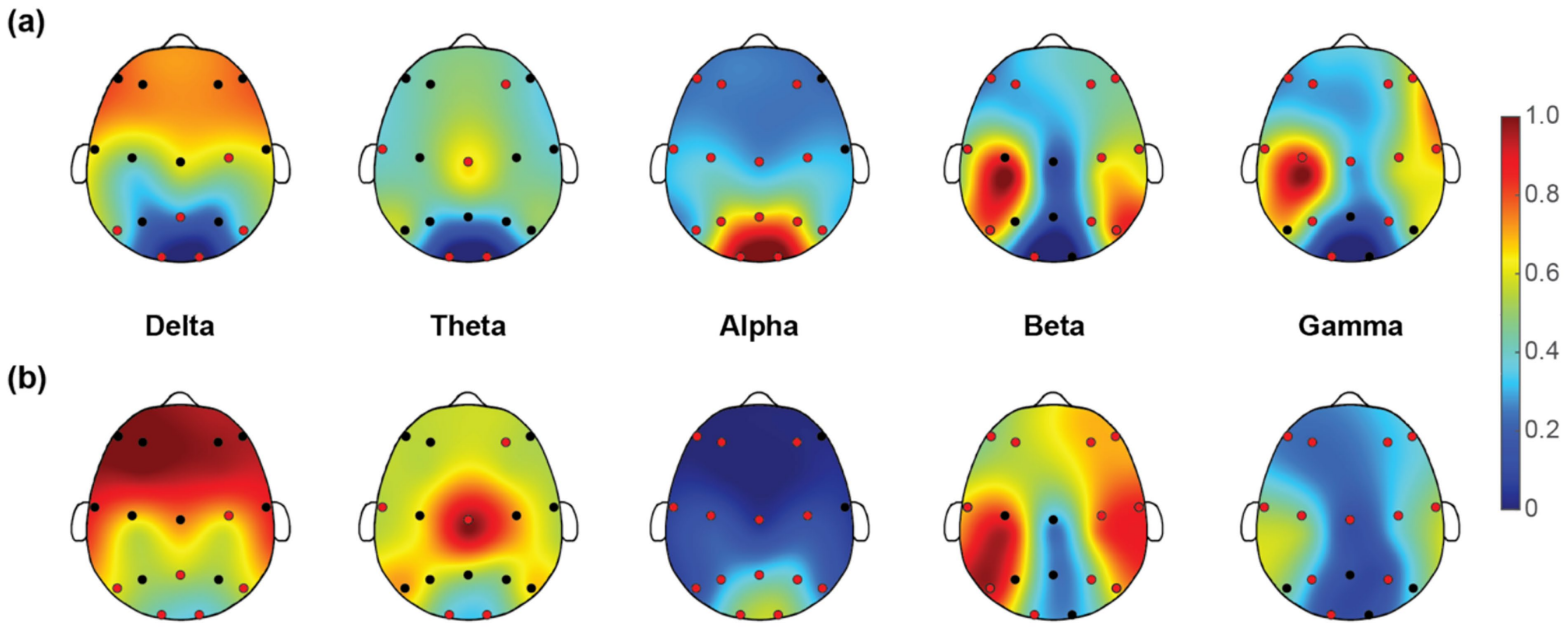
Topographic maps of relative power (*RP*) for five EEG rhythms in **(a)** HC and **(b)** pediatric SCZ groups. The red dots indicate the channels selected in the optimal feature subset.

**Figure 4:**
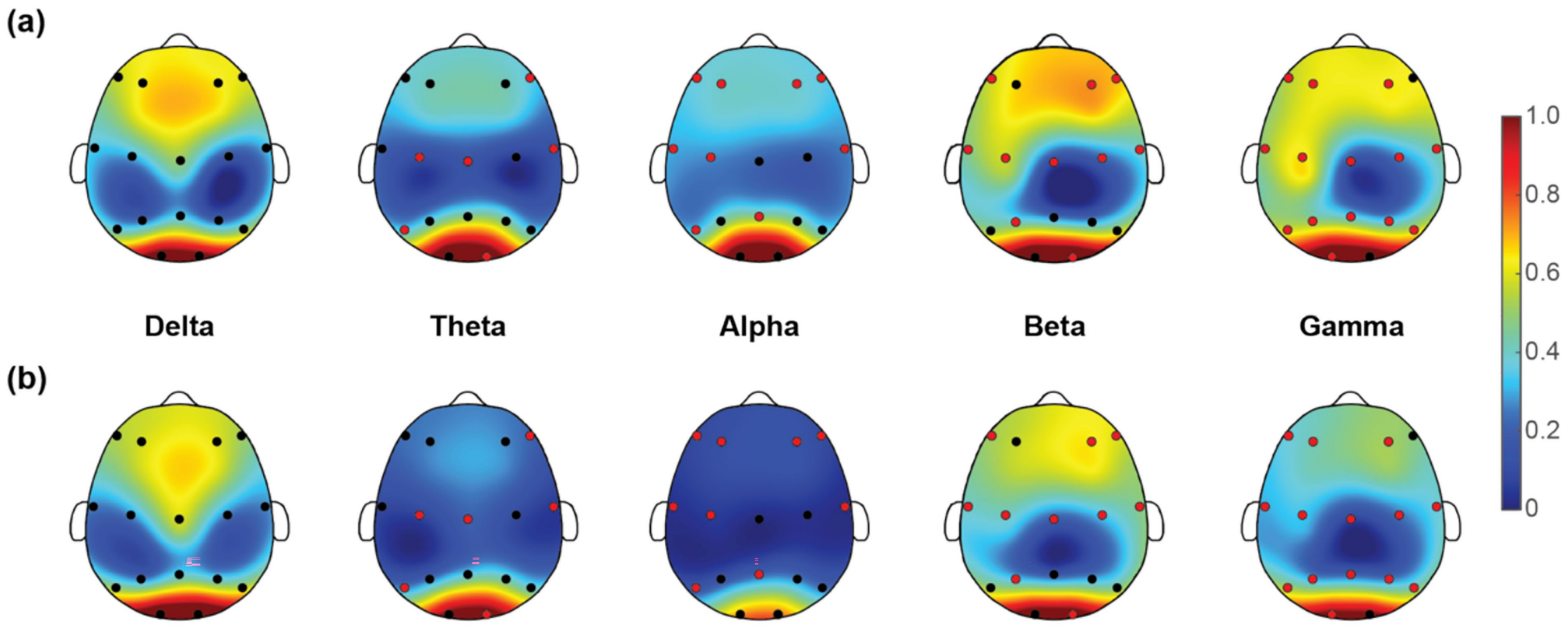
Topographic maps of fuzzy entropy (*FuzEn*) for five frequency bands in **(a)** HC and **(b)** pediatric SCZ groups. The red dots indicate the channels selected in the optimal feature subset.

**Figure 5:**
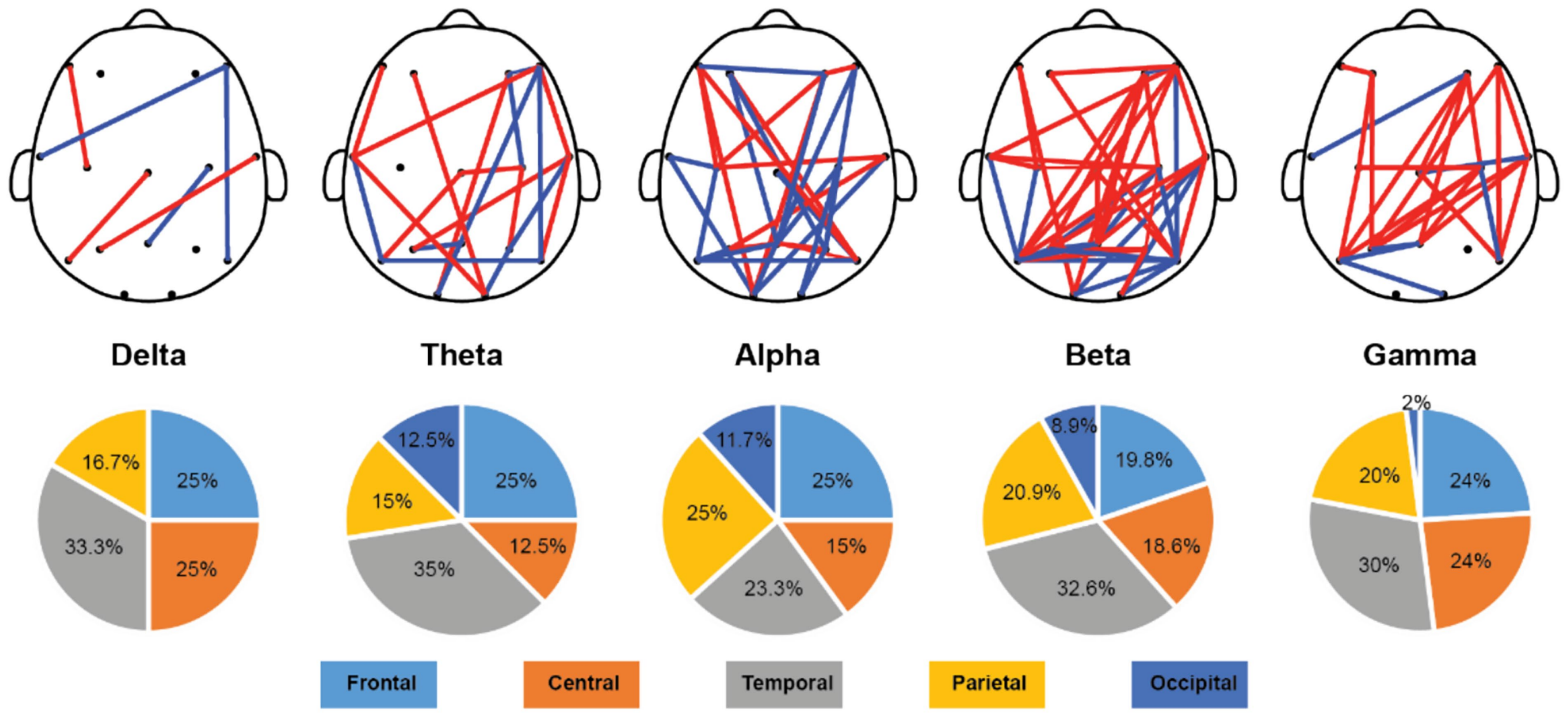
Topological distribution of the *PLI* features and the spatial distribution pie plot over five frequency bands. Red edges indicate that the *PLI* values for pediatric SCZ are higher than those for the HC group, while blue edges represent that the *PLI* values for pediatric SCZ are lower than those for the HC group.

Once we have obtained the optimal feature subset, we then look into the spatio-spectral distribution of the discriminative features separately. The brain topographic maps of relative power features are presented in Figure 3. Specifically, an increase of RP in delta, theta and beta and a decrease in alpha and gamma band were observed in pediatric SCZ patients. The regions with differences in delta were mainly distributed in frontal, temporal, and occipital areas, the regions with differences in theta were mainly distributed in central and occipital areas, whereas the regions with differences in beta, alpha, and gamma were spread across the entire brain. In Figure 4, we showed the topographic maps of *FuzEn* in both groups. For pediatric SCZ patients, a universal decrease pattern was observed in four frequency bands. No discriminative *FuzEn* feature was revealed in the Delta band. In terms of the spatial distribution, we found a fronto-central predilection in Theta, Alpha and Beta bands, while spread across the brain in the Gamma band. The *PLI* distribution results, as well as the corresponding proportion of each brain region are depicted in Figure 5. The research findings a predominantly increased *PLI* pattern was revealed in Theta, Beta and Gamma bands, linking frontal, central and parietal areas, where a decreased *PLI* pattern was found in the Alpha band, linking frontal, parietal and occipital regions with a rightward predilection.

## 4 Discussion

This study has established an innovative analytical framework, which relies on a multi-dimensional EEG feature set determined by the optimal time window length. By integrating ensemble learning models with feature selection algorithms, the framework aims to extract EEG features that contribute most significantly to the classification accuracy of pediatric SCZ and exhibit the most pronounced differences, thereby providing deeper insights into the brain functional mechanisms of pediatric SCZ patients. The main findings are as follows: (1) Satisfactory classification performance is achieved through incorporating multidimensional EEG features with ensemble learning models, and reaches the best performance using the CatBoost model under 4-s time window (classification accuracy = 99.60%). (2) Based on the analysis of the optimal feature subset corresponding to the highest accuracy, we investigate the spatio-spectral distribution and find pediatric SCZ is characterized as a complex dysconnectivity pattern mainly in the alpha, beta and gamma bands. This dysconnectivity pattern is accompanied by abnormal distributions of relative power and fuzzy entropy features in specific frequency bands. These findings will be discussed in detail below.

### 4.1 Classification performance of Pediatric SCZ

Due to the high temporal resolution of EEG signals, the corresponding time window length for feature extraction would inevitably influence the performance of the machine learning framework. The determination of optimal time window length has long been a popular research topic in recent SCZ classification studies. However, complex findings were reported in determining the optimal time window length. For instance, Shen et al. introduced a deep learning framework to identify SCZ using the same publicly available dataset (Shen et al., 2023). Specifically, EEG dynamic functional connectivity features were extracted with different time-window lengths (i.e., 2, 5, 10, 30 s) before a 3D convolutional neural network. They reported that a monotonic increasing trend of classification performance was obtained with the increase of time window length (from 80.13% in a 2-s window to 97.74% in a 30-s window) (Shen et al., 2023). However, a relatively short time window (1 s with 50% overlapping) was used on the same dataset to compute the effective FC features and achieved a satisfactory classification accuracy of 91.69% (Phang et al., 2020). The short time window (0.1–0.6 s post stimulus onset) was also adopted in a recent study and led to a classification performance of 95.15%. In exploring the influence of EEG time window on the classification performance of pediatric SCZ, we reveal the crucial role of time window length through rigorous experimental design and multi-dimensional EEG feature extraction. We found that the classification performance was saturated when the time window length was higher than 2 s and reached the best performance at 4 s (accuracy = 99.21%). The discrepancies could stem from the following two aspects: feature extraction methods and experimental design (resting-state *vs.* task design). Collectively, these studies underscore the criticality of time window selection in EEG signal processing and highlight the importance of optimizing time window length for improving classification performance.

The satisfactory recognition accuracy achieved in this study with a 4-s time window is attributed to the combined application of the CatBoost ensemble learning algorithm and feature selection algorithm. As an advanced ensemble learning method, the CatBoost algorithm excels in handling high-dimensional data and imbalanced datasets. The findings in this study reaffirm its effectiveness in complex EEG signal analysis. Meanwhile, the introduction of the feature selection algorithm, by eliminating redundant and irrelevant features, retains the most discriminative EEG features, thereby significantly enhancing classifier performance. This finding aligns with other research, which similarly achieved a significant improvement in SCZ patient classification accuracy through Bayesian optimization for selecting the best machine learning model and hyperparameters (Keihani et al., 2022). Notably, this study complements the research by Soria et al. They compared different machine learning systems and found that the ensemble learning algorithm performed well in SCZ classification with an accuracy of 94% (Soria et al., 2023). Therefore, these findings not only provide powerful technical support for the early diagnosis and treatment of SCZ but also offer new research ideas and methodological guidance for future EEG signal analysis in mental disorders, which may benefit the future development of automatic diagnosis systems. Of note, we have also compared the classification performance of the current work with several most recent studies using the same publicly available dataset (Table 3). In comparison with these previous studies, where fine-tuning neural network structure was utilized on single-domain EEG features, a lightweight ensemble learning model was adopted that delivers satisfactory performance (2^nd^ best). We believe the rich information embedded in the EEG signals could be extracted from multiple domains that would lead to a comprehensive understanding of the etiology of pediatric SCZ.

**Table 3:** Comparison of the best performance in the current work and recent studies in SCZ classification using the same dataset.

References	Features + Models	Accuracy	Sensitivity	Specificity
Aslan and Akin (2020)	STFT + VGG-16 CNN	95	95.37	94.68
Phang et al. (2020)	PDC + Multi-domain connectome CNN	91.69 ± 4.67	91.11 ± 8.31	89.64 ± 9.48
Sairamya et al. (2022)	DWT + relaxed local neighbor difference pattern + ANN	100	–	–
Shen et al. (2023)	Dynamic FC + 3D CNN	97.74 ± 1.15	96.91 ± 2.76	98.53 ± 1.97
Current work	Multidimensional EEG features (RP, FuzEn, PLI) + CatBoost	99.21 ± 0.50	98.98 ± 0.83	99.43 ± 0.62

STFT, short-time Fourier transform; CNN, convolutional neural network; PDC, partial directed coherence; DWT, discrete wavelet transform; ANN, artificial neural network; FC, functional connectivity; RP, relative power; FuzEn, fuzzy entropy; PLI, phase lag index.

### 4.2 Spatio-spectral distribution of the Most discriminative features

In order to explore the characteristics of EEG signals in pediatric SCZ patients, this study introduced a data-driven framework through incorporating ensemble learning models and a feature selection approach. Compared to the previous studies with complex deep learning or neural network structures, the framework provides direct correspondence with EEG characteristics with interpretable neurophysiological meanings. Specifically, pediatric SCZ patients exhibit complex alteration patterns across different frequency bands (i.e., an increase in delta, theta, and beta bands, while a decrease in alpha and gamma bands). This finding was in line with previous studies (Iglesias-Tejedor et al., 2022; Light et al., 2006; Zhang et al., 2021). This power change pattern is particularly prominent in the temporal and occipital regions, suggesting that these regions may play crucial roles in the pathological process of SCZ. Given the important role of the temporal region in memory, emotion and auditory processing and the occipital regions in visual processing, these alterations may represent a disrupted brain dynamic oscillation that may lead to the well-known hallucination and delusion symptoms. Moreover, a universal decrease pattern was revealed in fuzzy entropy in theta, alpha, beta, and gamma bands, indicating a less complex and unpredictable nature of EEG signals in patients. This finding was in line with a recent work, where Molina and colleagues reported deficits in spectral entropy modulation in patients with chronic and first-episode SCZ (Molina et al., 2020). In terms of brain network alterations/reorganization, the current work employed advanced methods to conduct in-depth research on functional connectivity across the whole brain in SCZ patients. Specifically, a widely-used *PLI* was adopted here to estimate the functional connectivity for its superiority in attenuating the influence of EEG volume conduction and common sources (Stam et al., 2007), leading to the intrinsic functional interactions. Among the most discriminative features, over half of them are *PLI* features (124 out of 212), indicating that SCZ is related to aberrant connectivity between distinct brain regions rather than abnormalities within the separate regions themselves. Moreover, we found a complex and widespread dysconnectivity pattern across five frequency bands in pediatric SCZ patients. It is noteworthy that the results of this study are highly consistent with those of previous brain connectome studies (Koshiyama et al., 2020). Contemporary theories suggest SCZ as a disorder of brain dysconnectivity or a disorder of brain network organization (Sheffield and Barch, 2016; Bassett et al., 2012). Our observations therefore extend previous studies of chronic and/or first-episode SCZ in adults to pediatric SCZ and provide further evidence for the notion of SCZ as a disconnection syndrome. Collectively, through a comprehensive analysis of relative power, fuzzy entropy, and functional connectivity features of EEG signals in SCZ patients, this study has revealed functional connectivity reorganization across brain regions and frequency bands, as well as abnormal distributions of relative power and fuzzy entropy features in specific frequency bands. These findings not only provide important clues for understanding the neurophysiological basis of pediatric SCZ but also offer potential biomarkers for future diagnosis and treatment.

### 4.3 Future considerations

Some issues should be considered when interpreting our findings. First, a widely-used publicly available dataset was used in the current work that includes 84 participants (pediatric SCZ/HC = 45/39). The relatively small sample size and the inclusion of only male participants may limit the generalizability and reproducibility of our findings. Evidence of gender differences in the brain and neurocognitive function in SCZ has long been recognized (Mendrek and Mancini-Marie, 2016). We opted for this choice to maximize the number of existing classification studies with which our results could be directly compared without the need to consider the influences of clinical and demographic differences between different datasets. Nevertheless, further studies with a larger independent study sample and the inclusion of both genders are recommended to confirm our observations. Second, a heterogeneous group of patients was recruited in the current work that includes infant SCZ, schizotypal and schizoaffective disorders. It is noteworthy that the heterogeneous phenotype of SCZ patients might be a potential influence in extracting EEG features that contribute to classification due to divergent neurophysiological mechanisms. Future research should delve deeper into the brain function characteristics of pediatric SCZ subtypes to provide more specialized strategies to assist in automatic diagnosis.

## 5 Conclusion

This study proposes an analytical framework that leverages multidimensional EEG features combined with ensemble learning models and feature selection algorithms, to identify the most discriminative EEG features between the pediatric SCZ and HC groups, ultimately revealing unique brain functional alterations in pediatric SCZ patients. The results indicated that the CatBoost algorithm achieved a 99.21% accuracy in identifying pediatric SCZ patients. Additionally, 212 most discriminative features were screened from a total of 760 features, constituting a key subset for pediatric SCZ recognition. Further analysis of the optimal feature subset revealed that pediatric SCZ patients exhibited complex dysconnectivity architecture accompanied by abnormal distributions of relative power and fuzzy entropy features in specific frequency bands. The findings of this study not only improved the accuracy of pediatric SCZ identification but also provided potential biomarkers for the automatic diagnosis of pediatric SCZ.

## Data Availability

The datasets presented in this study can be found in online repositories. The names of the repository/repositories and accession number(s) can be found below: http://brain.bio.msu.ru/eeg_schizophrenia.htm.
